# Supplementary figures and images for: Nanograms of SARS-CoV-2 spike protein delivered by exosomes induce potent neutralization of both delta and omicron variants
Source: PLoS One. 2023 Aug 22;18(8):e0290046. doi: 10.1371/journal.pone.0290046 (PMC10443850; doi:10.1371/journal.pone.0290046)

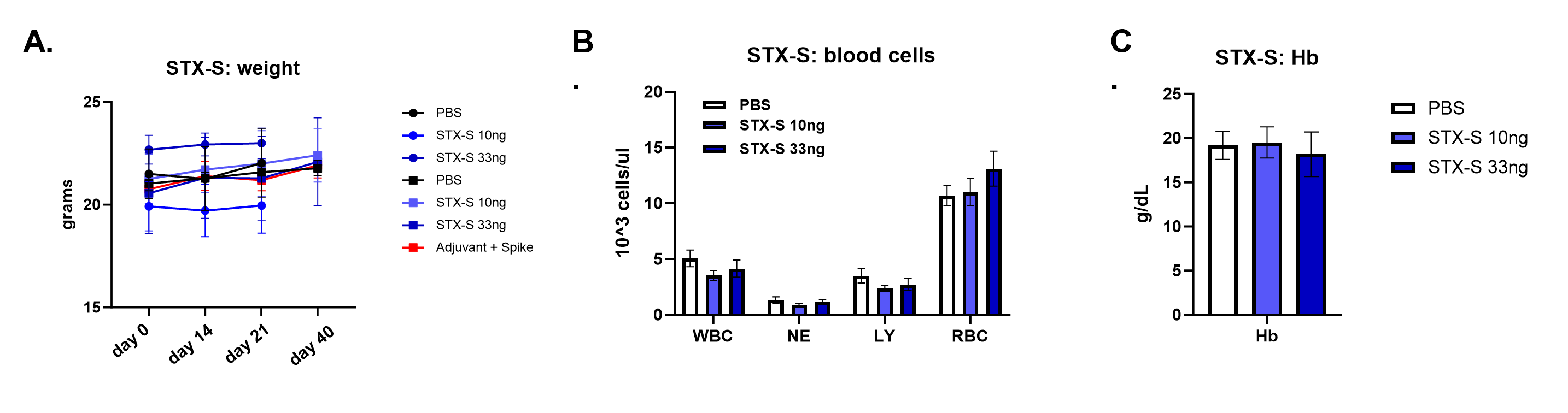

Supplement: S1 Fig — (TIF) [file pone.0290046.s001.tif]

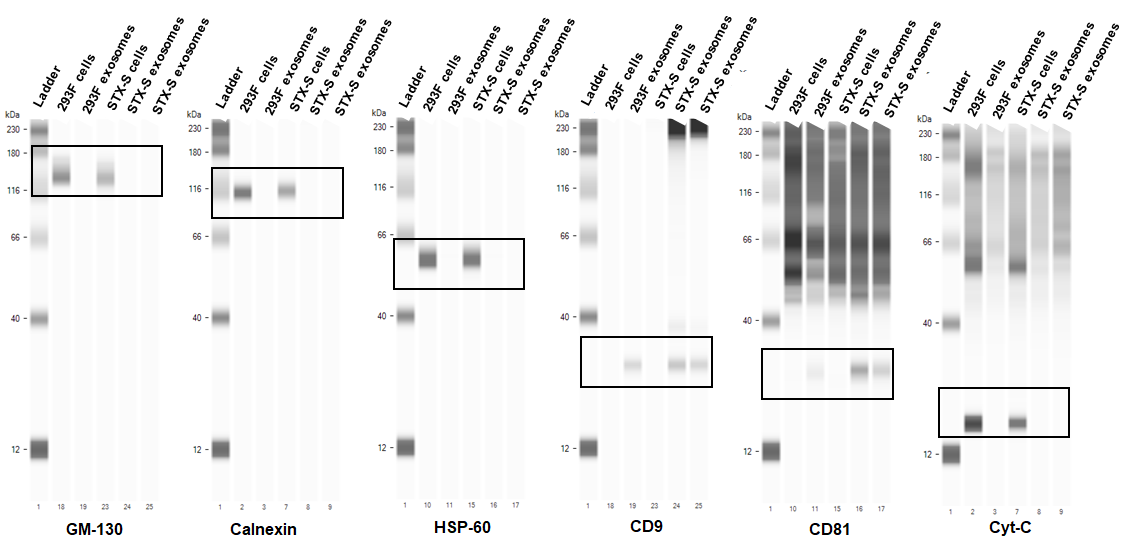

Supplement: S2 Fig — (TIF) [file pone.0290046.s002.tif]

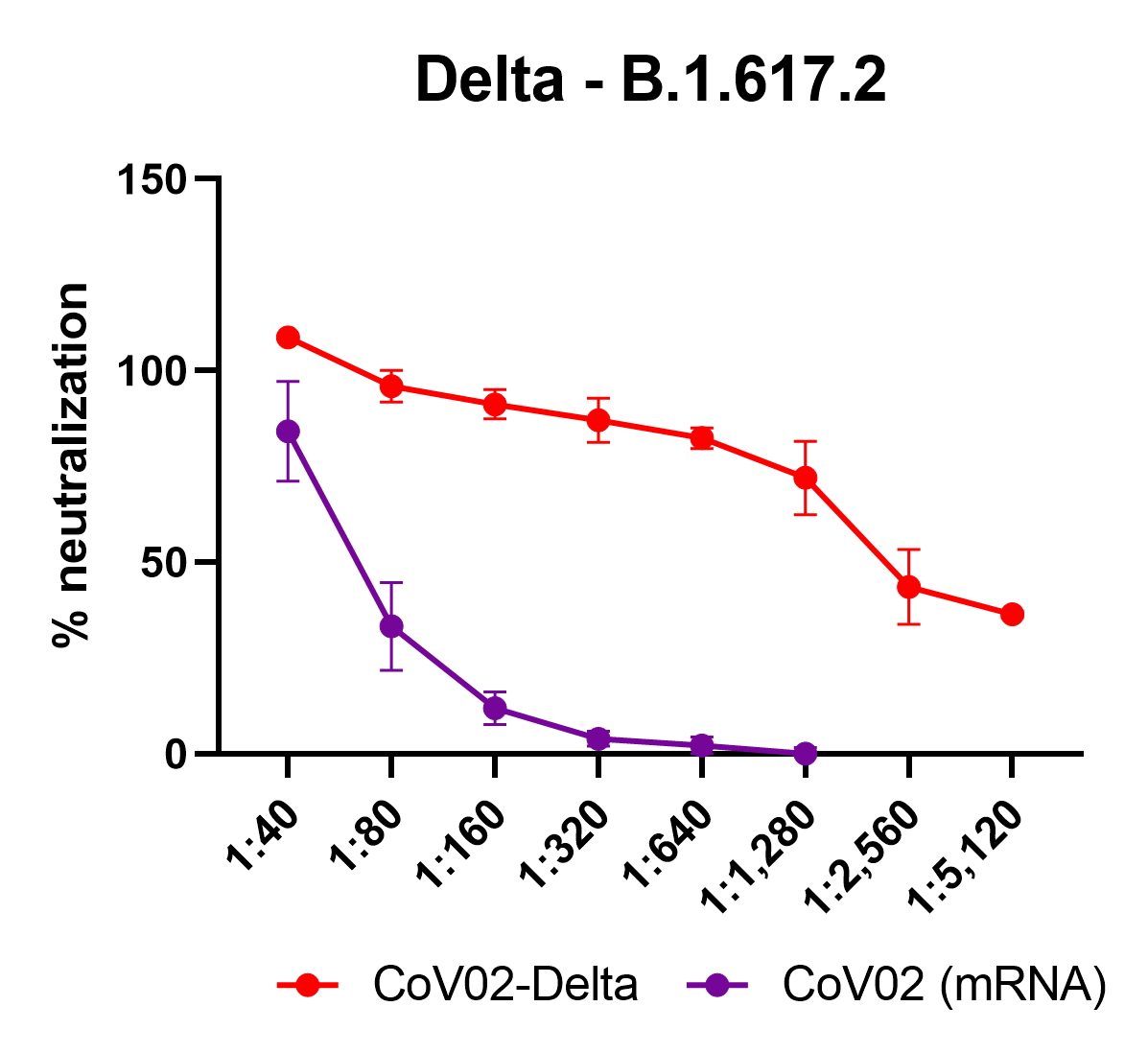

Supplement: S3 Fig — (TIF) [file pone.0290046.s003.tif]
