# Supplementary material for: Nanograms of SARS-CoV-2 spike protein delivered by exosomes induce potent neutralization of both delta and omicron variants
Source: PLoS One. 2023 Aug 22;18(8):e0290046. doi: 10.1371/journal.pone.0290046 (PMC10443850; doi:10.1371/journal.pone.0290046)

Original western blot, obtained using Jess Automated Western Blot, used to generate panel 2D in Fig. 2 of the manuscript. X represents additional samples not part of the study.

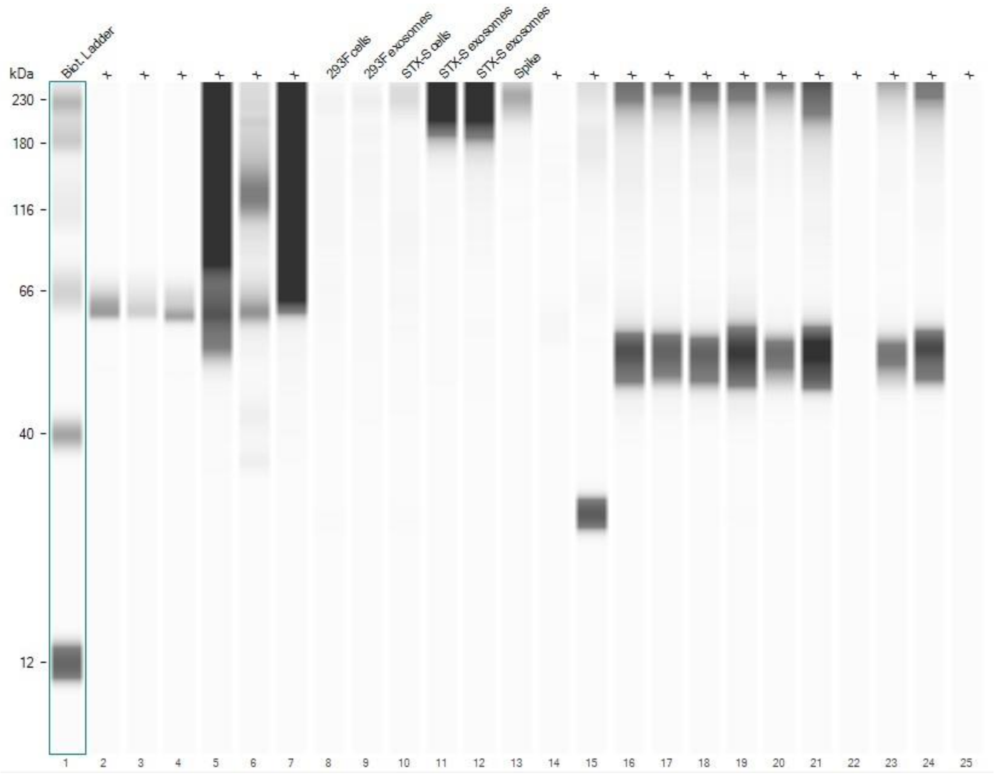

Original western blot, obtained using Jess Automated Western Blot, used to generate Supplementary Figure 2. X represents additional samples not part of the study. Please, refer to Supplementary figure 2 for a clear identification of samples and target.

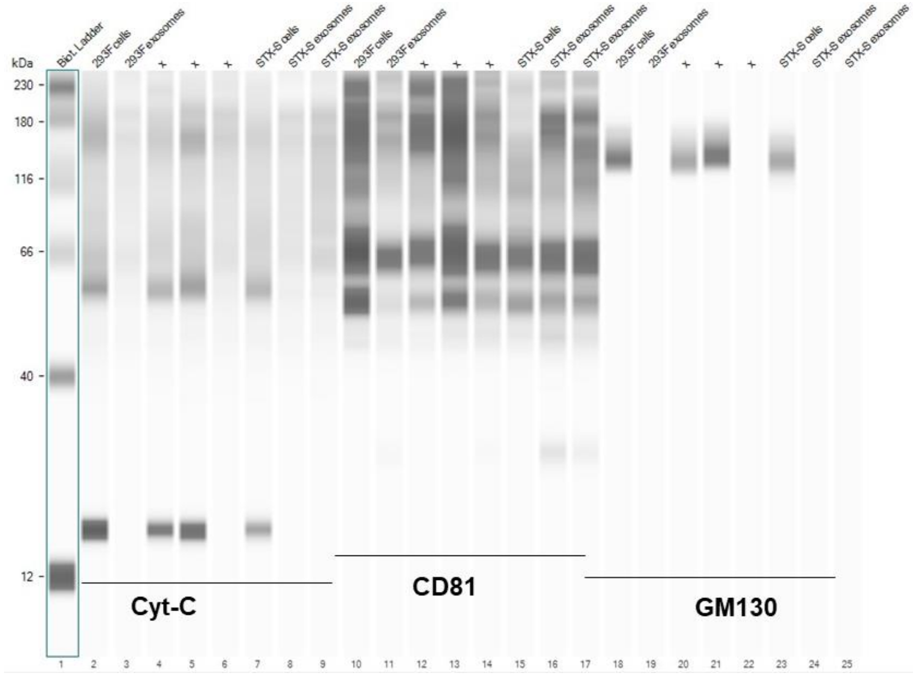

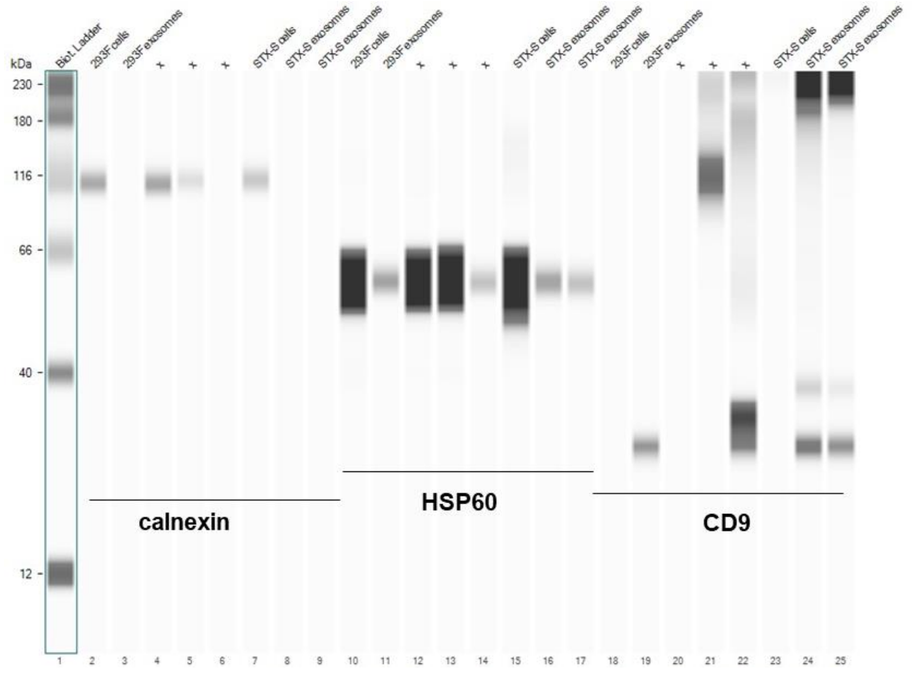

Supplement: S1 Raw images — (PDF) [file pone.0290046.s004.pdf]
