# Supplementary material for: Nanograms of SARS-CoV-2 spike protein delivered by exosomes induce potent neutralization of both delta and omicron variants
Source: PLoS One. 2023 Aug 22;18(8):e0290046. doi: 10.1371/journal.pone.0290046 (PMC10443850; doi:10.1371/journal.pone.0290046)

Original western blot, obtained using Jess Automated Western Blot, used to generate panel 2D in Fig. 2 of the manuscript. X represents additional samples not part of the study.

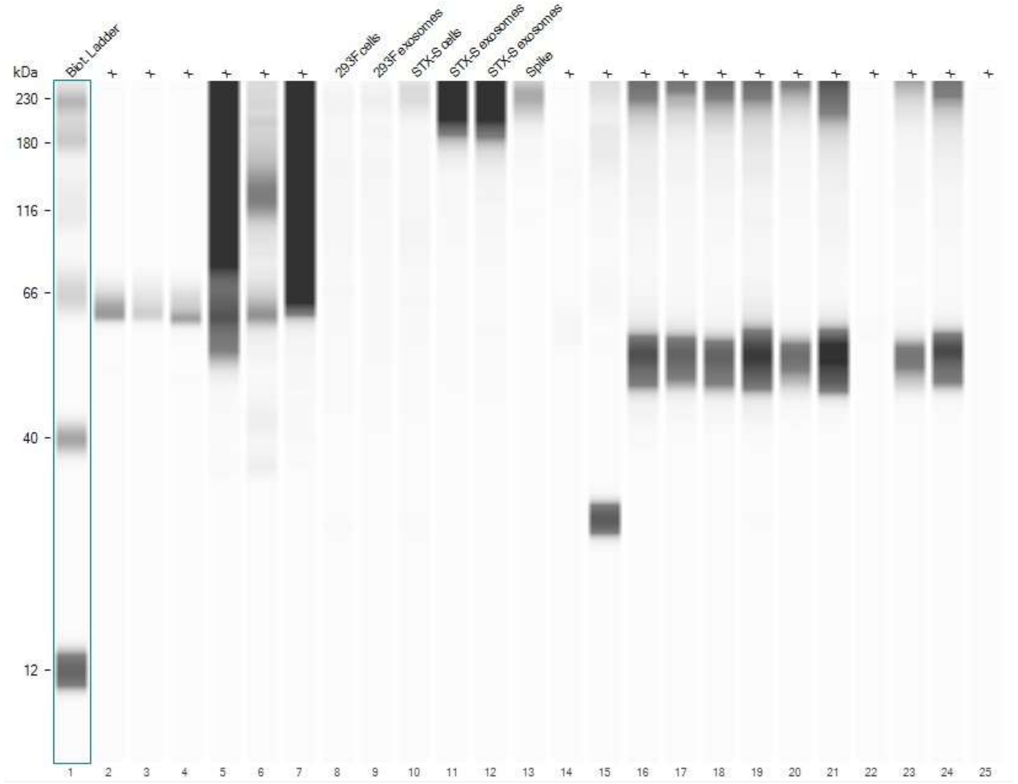

Supplement: S2 Raw images — (PDF) [file pone.0290046.s005.pdf]
